# Clinical, Laboratory, and Treatment Profiles of Silent Corticotroph Adenomas That Have Transformed to the Functional Type: A Case Series With a Literature Review

**DOI:** 10.3389/fendo.2020.558593

**Published:** 2020-09-23

**Authors:** Guangyao Zheng, Lin Lu, Huijuan Zhu, Hui You, Ming Feng, Xiaohai Liu, Congxin Dai, Yong Yao, Renzhi Wang, Huabing Zhang, Xu Sun, Zhaolin Lu

**Affiliations:** ^1^Key Laboratory of Endocrinology of National Health Commission, Department of Endocrinology, Peking Union Medical College Hospital, Chinese Academy of Medical Science and Peking Union Medical College, Beijing, China; ^2^School of Clinical Medicine, Weifang Medical University, Weifang, China; ^3^Department of Radiology, Peking Union Medical College Hospital, Chinese Academy of Medical Science and Peking Union Medical College, Beijing, China; ^4^Department of Neurosurgery, Peking Union Medical College Hospital, Chinese Academy of Medical Science and Peking Union Medical College, Beijing, China

**Keywords:** treatment, diagnosis, cushing syndrome, silent corticotroph adenoma, nonfunctional pituitary adenoma, temozolomide

## Abstract

**Purpose:** Silent corticotroph adenoma (SCA) is clinically non-functional pituitary adenoma with expression of corticotropin or Tpit. To further understand the characteristics of this rare type of SCA transforming to a functional SCA, we retrospectively reviewed SCAs that converted to typical Cushing's syndrome at a tertiary medical center and the relevant literature.

**Methods:** Patients were identified based on the diagnosis of pituitary adenoma without symptoms of hypercortisolism at the initial visit with positive Immunohistochemical (IHC) staining for corticotropin or Tpit after surgery and subsequent transformation to functional SCAs during the follow-up period from March 1990 to January 2020 at Peking Union Medical College Hospital and in the literature. The characteristics of the clinical manifestations, biochemical results, imaging findings, pathology findings and outcome were analyzed.

**Results:** Altogether, 16 patients were included in the study with an average age of 42.0 ± 12.48 (18–65) years at the first visit. Females were slightly predominant (F:M = 1.3:1). The median time of conversion from the nonfunctional to the functional type was 30 (13.0, 68.3) months. Once a functional SCA developed, the adrenocorticotropic hormone (ACTH) level and 24-h urine free cortisol were increased 3.8- (2.6, 12.9) and 5.3- (2.6, 19.3) fold, respectively, above the normal range. Approximately 50% of the patients had macrocystic changes on pituitary MRI. All 16 patients experienced 1–5 surgeries with a median of 2.5 (2.0, 4.0) surgeries. The proportion of patients with Ki-67 ≥ 3% increased from 22.2% (2/9) at the beginning to 50% (7/14) at the time of functional SCA diagnosis. Thirteen patients received radiotherapy, and 4 patients (30.8%) achieved remission. Four patients with refractory functional SCAs received temozolomide treatment with the normalization of cortisol in 4 cases and reduced tumor volume in 3 cases.

**Conclusion:** In this study, all cases that transformed to functional SCAs were macroadenomas. Hypercortisolism was more severe in functional SCA patients. The tumors tended to have frequent recurrence and were highly invasive. Temozolomide could be a promising treatment for refractory functional SCA cases. Long-term follow-up is needed for nonfunctional SCAs since some cases have the potential to transform to clinical Cushing's syndrome.

## Introduction

Pituitary adenomas originate from the pituitary gland and are slow-growing and benign in most cases. An autopsy series reported that the prevalence could be as high as 10–15%, accounting for ~15–20% of intracranial tumors (1–5). A study showed that the prevalence of pituitary adenomas in 2012 was 115.57/100, 000 in a surgical series (6). Pituitary adenomas can be classified into non-functional adenomas and functional adenomas according to the presence or absence of hormone overproduction. The most common type of pituitary adenoma is a non-functional pituitary adenoma (NFPA), 20% of which might not be diagnosed due to the absence of typical symptoms (7, 8). In epidemiological data from Iceland between 1955 and 2012, the relative prevalence of NFPAs was 42.32 per 100,000 in a surgical series. NFPAs were the most common (43.0%) pituitary adenomas, followed by prolactinomas (39.9%), acromegaly (11.3%), and Cushing's disease (5.7%) (6). However, there were some special cases of “clinically silent but pathological active” pituitary adenomas among the NFPAs; they were called “silent pituitary adenomas,” which indicated that these pituitary adenomas had immunohistochemical (IHC) expression of one or more anterior pituitary hormones but no relevant clinical symptoms. Recently, it was recommended that, in addition to the expression of hormone levels, transcription factors should be measured to define the actual lineage of the tumor according to the 2017 WHO classification of pituitary adenomas. Nishioka et al. (9) investigated the complementary role of transcription factors in consecutive NFPAs (*n* = 516). Depending on the IHC findings for hormones, these NFPAs could be classified as follows: gonadotroph adenomas (58.1%), hormone-negative adenomas (*n* = 119, 23.1%), silent corticotroph adenomas (SCAs) (9.9%), and 46 other silent adenomas. Further evaluation of hormone-negative adenomas showed that 95% (*n* = 116) of those tumors expressed transcription factors as follows: gonadotroph adenomas (SF-1 positive, 66.4%), SCAs (Tpit positive, 26.9%), etc. Thus, the SCAs, distinguished by integrating IHC staining for hormones with transcription factors, were the second most common (16.1%, 83/516) of the above mentioned clinically silent NFPAs.

The first SCA case in the literature was reported by Kovacs et al. (10). A 43-year-old man diagnosed with NFPA presented with positive adrenocorticotropic hormone (ACTH) expression in the tumor tissue without symptoms of hypercortisolism, increased cortisol, or ACTH levels. It is commonly known that SCAs have no clinical manifestations of Cushing's syndrome, but they are highly aggressive and often invade the cavernous and sphenoid sinuses. Histologically, SCAs can be divided into three subtypes: densely granulated corticotroph adenomas, sparsely granulated corticotroph adenomas, and Crooke cell adenoma (11). It is worth noting that the first case of NFPA that transformed into a functional corticotroph adenoma, after 7 years of follow-up, was reported by Cooper et al. (12), causing some concern among clinicians. Subsequently, a few cases of SCAs that transformed from silent to clinically functional corticotroph adenomas have been reported (13–18). However, the underlying mechanism of the transformation of function has not yet been elucidated.

To further explore the characteristics of transformation from non-functional to functional SCA types, which may have clinical significance for the management of these patients, patients with SCAs who underwent transformation were identified from the Peking Union Medical College Hospital records and the published literature. The clinical manifestations, biochemical tests, imaging features, and IHC features of these cases were further analyzed.

## Methods

### Subjects

The clinical data of patients who were initially diagnosed with NFPAs and presented later with typical Cushing's syndrome and abnormal hormone tests during long-term follow-up were collected at Peking Union Medical College Hospital from March 1990 to December 2019. In addition, we reviewed the literature (from PubMed) about SCAs with the transformation from non-functional to functional types.

### Silent Corticotroph Adenoma Cases That Transformed Into Functional Corticotroph Adenomas at Peking Union Medical College Hospital

The patients' data were obtained through a search of the hospital information system at Peking Union Medical College Hospital from 1990 to January 2020. The Ethics Committee of Peking Union Medical College Hospital approved this retrospective study.

The inclusion criteria included the following: (1) patients with pituitary adenomas who underwent surgery, (2) pituitary adenoma's that were totally silent without typical manifestations of Cushing's syndrome at the first visit, (3) no tests indicating hypercortisolism at the first visit (normal ACTH, 24-h urinary free cortisol [UFC], and midnight cortisol or suppressed low-dose dexamethasone suppression test [DST] if ACTH or cortisol was elevated), (4) positive IHC staining for ACTH and/or Tpit on the first or subsequent pathological results after surgery, (5) gradual appearance of the typical clinical manifestations of Cushing's syndrome and the laboratory examination of ACTH-dependent Cushing's syndrome (normal or increased ACTH, increased midnight cortisol or 24-h UFC, no suppression on the low-dose DST) during the postoperative follow-up period, and (6) detailed medical information that could be collected. The exclusion criteria included the following: (1) abnormality on at least one of the mentioned tests for hypercortisolism at the first endocrine evaluation (despite no clinical manifestations of hypercortisolism), (2) diagnosis of ectopic ACTH syndrome, (3) incomplete medical information, or (4) no findings of either ACTH or Tpit expression in tumor tissues on IHC. Finally, seven SCA patients initially diagnosed with NFPAs met the enumerated inclusion/exclusion criteria and had transformation to typical Cushing's syndrome during the follow-up period. We collected information about these cases, including (1) demographic features such as age and sex; (2) the time of appearance of Cushing's syndrome; (3) clinical symptoms; in particular, clinical manifestations relevant to Cushing's syndrome including central obesity, hypertension, purple striae, buffalo hump, weight gain, skin thinning, moon face, osteoporosis, hypokalemia, and hyperglycemia; (4) biochemical tests including blood glucose, serum potassium, and blood lipids; (5) hormonal tests including ACTH, serum cortisol, 24-h UFC, and DST; (6) results of enhanced pituitary magnetic resonance imaging (MRI); (7) pathological results and IHC staining for pituitary hormones, and transcription factors; and (7) details of treatment.

The diagnostic procedure for Cushing's syndrome depends on the typical symptoms and signs, including increased midnight cortisol (>1.8 μg/dl), 24-h UFC, and no suppression on the low-dose DST (including overnight DST or 2 mg/day 48-h low-dose DST). The 48-h, 8-mg/day high-dose DST was also carried out for the patients admitted to our center. Suppression rates above 50% after the high-dose DST indicated suppression. Because all the patients had macroadenomas, International Prostate Symptom Score was not necessary.

### Literature Review of Silent Corticotroph Adenoma Cases That Transformed Into Functional Corticotroph Adenomas

We conducted a literature search of the PubMed online database through February 1, 2020; we searched for keywords including [Cushing's syndrome] AND [SCA] OR [silent ACTH adenomas] OR [silent corticotroph adenomas] OR [non-functional pituitary adenomas] OR [silent pituitary adenomas]. The inclusion criteria were the same as those mentioned, and the literature exclusion criteria were as follows: (1) there was no transformation to Cushing's syndrome in the description; (2) cases could not be diagnosed as SCA according to the pathological IHC staining; (3) cases were not reported in English; (4) the literature had already been reviewed, and there were no new cases; or (5) there was a lack of detailed data on the clinical manifestations and biochemical tests related to this study. Finally, 65 studies were identified, 13 of which described the transformation from “silent” SCA to functional corticotroph adenoma, and 6 of them had detailed data. Altogether, nine cases from six studies met the inclusion/exclusion criteria, and relevant medical information was collected.

### Statistical Methods

Qualitative data are presented as frequencies and percentages. Quantitative data are presented as the means ± standard deviations or medians (ranges). If the data did not conform to the normal distribution, the median (25th percentile, 75th percentile) was used. Independent two-sample t-tests were used to evaluate the statistical significance. The significant differences in the data were analyzed using the Wilcoxon rank-sum test and the Kruskal–Wallis test. Statistical analysis was performed using SPSS version 19 (IBM, Armonk, New York). *P* < 0.05 indicated significance. A statistical chart was made with GraphPad Prism 7 (GraphPad Software, La Jolla, California, USA).

## Results

A total of 16 SCA patients who had a diagnosis of progressive change from non-functional to functional SCA were identified in our hospital (*n* = 7) and the literature (*n* = 9) (14, 16–18).

### Demographics

Among the 16 patients, there were seven males (43.8%) and nine females (56.2%) with a sex ratio (M:F) of 1:1.3. The average age at their first visit was 42.0 ± 12.5 years (18–65 years), 46.4 ± 14.3 years (18–65 years), and 38.9 ± 9.6 years (18–49 years) in all patients, male patients, and female patients, respectively. The average age at diagnosis of a clinically functional corticotroph adenoma was 45.7 ± 11.8 years (21–67 years), 48.6 ± 13.88 years (21–67 years), and 43.4 ± 9.25 years (24–54 years) in all patients, male patients, and female patients, respectively. The average age at the first visit and the diagnosis of typical Cushing's syndrome in males tended to be older than that in females, but there was no significant difference. The most common age group at the initial visit was 40–49 years (nine cases, 56.3%). The interval between the diagnosis of SCA at the initial visit to the diagnosis of overt Cushing's syndrome was 12–120 months, with a median of 30 (13.0, 68.3) months. The most common interval of transformation was 12–24 months in eight cases (50%).

### Clinical Manifestations

Among the 16 patients with SCA, none showed obvious relevant symptoms of Cushing's syndrome initially according to their medical charts and descriptions in the literature. At the first visit, the symptoms of the patients manifested as visual impairment (62.5%, 10/16), headache (12.5%, 2/16), and hypopituitarism (12.5%, 2/16) and incidentally discovered after traffic accident without symptoms (2/16, 12.5%). However, during the follow-up period, they developed several symptoms, including weight gain, purple striae, skin thinning, central obesity, buffalo hump, and hypertension. No information about hypertension or hyperlipidemia was reported in the literature. Therefore, it was only from the seven patients from our medical center that we could obtain the frequency of hypertension (57.1%, 4/7) and hyperlipidemia (42.9%, 3/7). In addition to symptoms of the Cushing's syndrome, there were other symptoms, such as blurred vision (43.8%, 7/16), defect of the visual field (25%, 4/16), ptosis of the eyelid (6.3%, 1/16), dizziness (6.3%, 1/16), headache (6.3%, 1/16), and lactation (6.3%, 1/16). These symptoms, which are unusual in common Cushing's disease, may have been caused by compression due to the large size of the tumors in these patients.

### Hormonal Tests

The main results of the hormonal tests after the transformation to functional corticotroph adenoma are shown in Table 1. Serum potassium was available in the seven patients from our medical center. They all had hypopotassemia, and the average potassium level was 2.6 ± 0.2 (2.3–3.0) mmol/L. The morning ACTH, cortisol, and 24-h UFC levels ranged between 21.5 and 1,500 pg/ml, 20.2–75 μg/dl, and 210–2,454.6 μg/24 h, respectively, in the 16 patients. Considering that the 16 cases were reported from different medical centers, the normal ranges for hormone tests were different. The fold increase above the upper limit of the normal range (ULN) was used to show the degree of increased ACTH, cortisol, and 24-h UFC. These patients showed variable elevation of 24-h UFC (3–23.7-fold increase) with a median 5.3-fold (2.6, 19.3) increase above the ULN. The morning serum cortisol was 1.7-fold (0.9- to 3.4-fold) above the ULN. The morning plasma ACTH levels were 1.7- to 14.5-fold above the ULN with a median 3.8-fold increase (2.6, 12.9). In all patients, the low-dose DST could not be suppressed. The high-dose DST was carried out in five patients, with four patients showing no suppression. The suppression rates of the high-dose DST were 41.6, −43.4, −72, and −33.7% in these four patients. It was unexpected that three patients had a paradoxical increase in 24-h UFC after the high-dose DST, which is more commonly observed in some cases of primary pigmented nodular adrenal dysplasia.

**Table 1:** Clinical features, imaging features, IHC features, and treatment of 16 patients with a transformation to functional SCA.

	**Cases from our center**								**Cases from the literature**							
	**Case 1**	**Case 2**	**Case 3**	**Case 4**	**Case 5**	**Case 6**	**Case 7**	**Case 8**	**Case 9**	**Case 10**	**Case 11**	**Case 12**	**Case 13**	**Case 14**	**Case 15**	**Case 16**
Sex	F	M	F	M	M	M	F	F	F	F	M	F	M	M	F	F
Age at first visit	42	43	31	42	62	46	33	47	18	37	49	49	65	18	45	48
Time between onset and functional SCA (months)	120	56	72	12	12	12	57	84	72	24	24	12	24	36	46	16
**Laboratory examination at the diagnosis of CS**																
Serum potassium (mmol/L)	2.8	2.7	2.5	2.3	2.6	3.0	2.6	NA	NA	NA	NA	NA	NA	NA	NA	NA
ACTH at 8 am (pg/ml) (NR: 0–46) (fold increase above the ULN)	119 (2.6)	361 (7.8)	201 (4.4)	76.5 (1.7)	669 (14.5)	376 (8.2)	173 (3.8)	1008^†^ (5–60) (16.8)	21.5 (0.5)	1,500^†^ (9–52) (28.8)	127.9^†^ (5–52) (2.5)	140^†^ (7.5–51.9) (2.7)	151.8 (3.3)	173 (3.8)	677 (14.7)	140(3)
Cortisol at 8 am (μg/dl) (NR: 4–22.3) (fold increase above the ULN)	20.2 (0.9)	75 (3.4)	39.7 (1.8)	47 (2.1)	54.8 (2.5)	55.9 (2.5)	47.3 (2.1)	34.1^‡^ (7–21) (1.6)	41.2^‡^ (5.6–23) (1.8)	44.5^‡^ (6.2–19.4) (2.3)	30.6^‡^ (7–25) (1.2)	26.8^‡^ (5–20) (1.3)	24.5 (1.1)	32.5^‡^ (5–25) (1.3)	23.2 (1)	21.6(1)
24-h UFC (μg/24 h) (NR: 12.3–103.5) (fold increase above the ULN)	312.3 (3)	2,367.4 (22.9)	1,261.7 (12.2)	2,454.6 (23.7)	623.6 (6)	1,981.3 (19.1)	2,072.8 (20)	240^*^ (35–137) (1.8)	210^*^ (20–90) (2.3)	630^*^ (36–137) (4.6)	1,307.5^*^ (50–190) (3.3)	NA	1,132.8 (10.9)	267 (1.4)	279 (2.7)	NA
**Imaging Features**																
Tumor size at the first visit (cm) (volume cm^3^)	4 × 4 × 2.5 (20.9)	1.5 × 1.5 × 2.1 (2.5)	4.1 × 4.7 × 2.9 (29.2)	2.1 × 1.6 × 2.3 (4)	2.3 × 1.3 × 1.1 (1.7)	3.5 × 3.3 × 3.1 (18.7)	NA	NA	NA	NA	NA	NA	NA	NA	NA	NA
Tumor size at the time of diagnosis of a functional SCA (cm) (volume cm^3^)	5.4 × 4 × 3.2 (35.5)	2 × 1.5 × 2 (3.2)	4.4 × 3.3 × 4 (30.4)	2.5 × 2.7 × 3 (10.6)	2.3 × 2 × 1.5 (3.6)	5.6 × 3.5 × 3 (30.8)	1.5 × 1. 9 × 2 (3)	NA	NA	NA	NA	NA	NA	NA	NA	NA
Tumor invasion	Cavernous+ sphenoid	Cavernous	Cavernous	None	Sphenoid	Cavernous	None	Cavernous	Cavernous	Cavernous	None	Cavernous	sphenoid	None	Cavernous + sphenoid	None
Cystic changes	Cystic degeneration	None	Macro cystic and microcystic	Macro cystic and microcystic	None	None	None	Macrocystic	Macrocystic	Macrocystic	Macrocystic	None	None	Macrocystic	NA	NA
**Ki-67 index in pathological IHC examination after surgery**																
At the first surgery	NA	NA	1%	1%	5%	NA	NA	2%	1%	2%	2%	NA	1%	NA	8%	NA
At the time of diagnosis of a functional SCA	1%	8%	1%	15%	5%	5%	20%	2%	2%	3%	2%	NA	1%	1%	12%	NA
**Specific Treatments**																
Surgery (number)	5	5	2	4	4	2	3	4	4	3	1	2	1	2	2	2
Radiotherapy (courses)	2	3	1	1	1	1	1	2	0	0	0	1	1	1	2	1
TMZ therapy	No	Yes	No	No	Yes	Yes	No	No	No	No	No	No	Yes	No	Yes	No
**Follow-up**																
Duration (months)	357	141	112	107	85	81	115	57	24	24	72	120	168	NA	48	228
Hormone remission^#^	Yes	Yes	Yes	No	Yes	Yes	Yes	No	No	No	Yes	No	Yes	No	Yes	No
Tumor reduction	Yes	Yes	Yes	No	No	Yes	Yes	Yes	Yes	Yes	Yes	Yes	Yes	No	Yes	Yes

*NR, normal range*.

ULN, upper limit of the normal range.

Tumor volume: calculated as (1/6πABC). A, B, and C are the length, height, and width of the tumor, respectively.

† *ACTH: Reference ranges for cases 8, 9, 10, 11, and 14 were different from the others, so they are listed separately*.

‡ *Serum cortisol: Reference ranges for cases 8, 9, 10, 11, 12, and 14 were different from the others, so they are listed separately*.

* *Urinary free cortisol: Reference ranges for cases 8, 9, 10, and 11 were different from others, so they are listed separately*.

### Imaging Features

**Figure 1:**
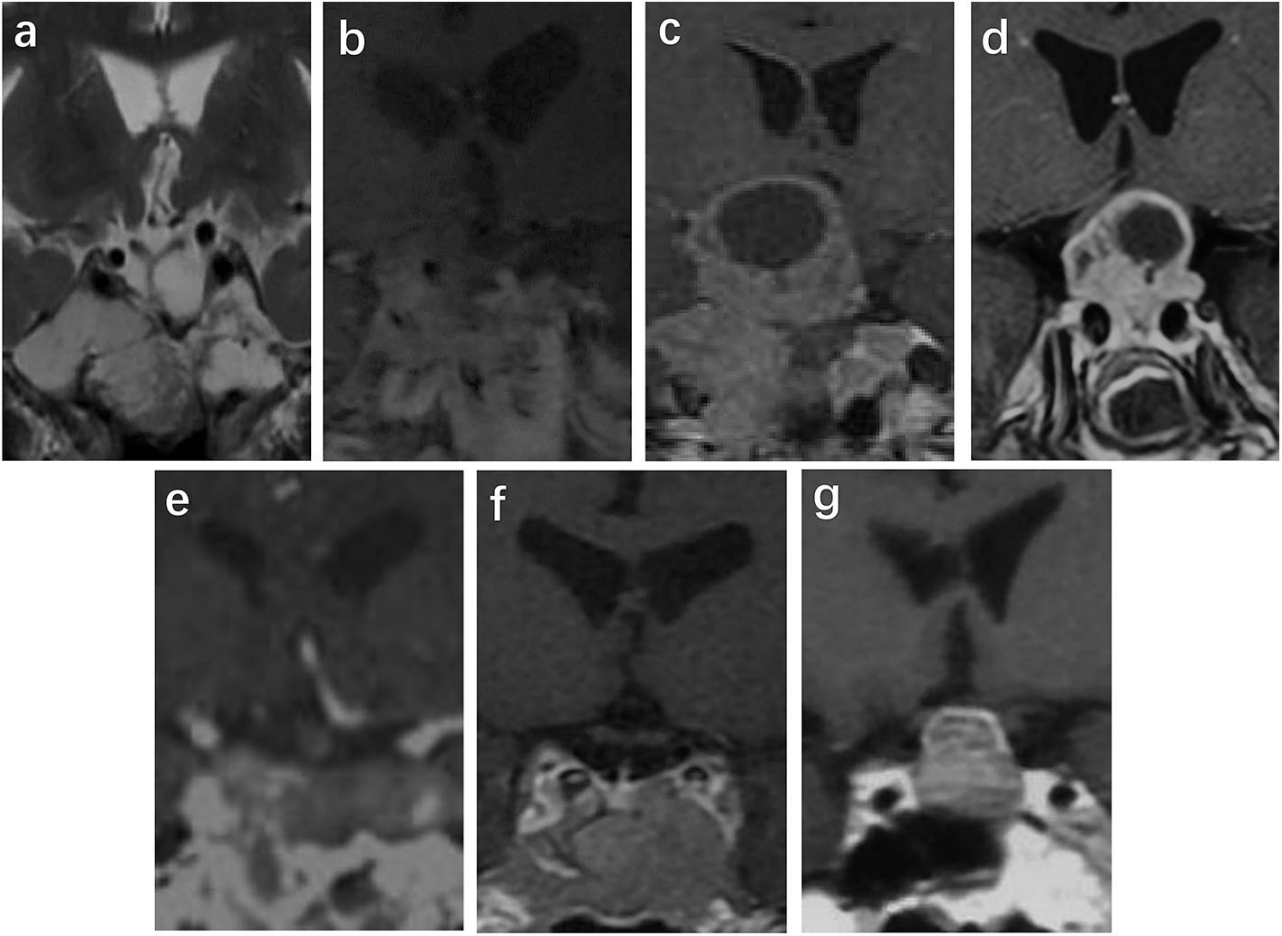
Images at the time of diagnosis of a functional SCA in cases 1–7 **(a–g)**. Sella MRI was performed in cases 1 **(a)**, 2 **(b)**, 3 **(c)**, 4 **(d)**, 6 **(f)**, and 7 **(g)**. Sella CT was performed in case 5 **(e)** because a pacemaker was installed. There is cavernous invasion in **(b)**, sphenoid sinus invasion in **(d)**, and both types of invasion in **(a)**. There are macrocystic changes and surrounding microcystic changes in **(b,c)**. According to T2-phase MRI of case 1 (a), the tumor tended to have cystic degeneration.

The MRI results were available in 15 patients, and CT results were available in one patient who had an implanted pacemaker at the time of functional SCA diagnosis; these imaging results were used to identify tumor invasion. All the tumors were macroadenomas. The invasion was detected as follows: cavernous sinus invasion in seven cases (43.8%) (17, 18), sphenoid sinus invasion in two cases (12.5%) (13), simultaneous sphenoid sinus and cavernous sinus invasion in two cases (12.5%) (14), and no sphenoid sinus or cavernous sinus invasion in five cases (31.3%) (15, 16). The MRI images from six cases and CT images from one case in our center are shown in Figure 1. Contrast MRI or CT images were obtained in 14 cases, and in 7/14 cases (50%), they demonstrated cystic changes (most of them were macrocystic changes). However, the size of the tumor could be obtained only for the seven cases from our medical center. The range of tumor volume was 1.7–29.2 cm^3^ with a median of 11.4 (2.3, 23.0) cm^3^ and maximal diameter of 2.1–4.7 cm at the first visit (*n* = 6), and increased to 3–35.5 cm^3^ with a median of 10.6 (3.2, 30.8) cm^3^ and maximal diameter of 2–5.6 cm at the time of diagnosis of functional SCAs (*n* = 7).

### Immunohistochemical Features on Pathological Examination

For the IHC staining of ACTH, although only eight patients (8/15) showed positive expression of ACTH and one patient did not have available information about ACTH staining after the first surgery, positive IHC staining for ACTH was found in all 16 patients at the time of diagnosis of Cushing's syndrome, which supported the diagnosis of SCA. For the IHC staining of transcription factors, Tpit staining was performed in only one case and showed positive results (case 2). Although the SCA tumors tended to be invasive and recurred easily, the range of IHC staining for the Ki-67 index was 1–8%, with a median of 2% (1%, 3.5%) after the first surgery (*n* = 9 patients with available data). However, the Ki-67 index had a wide range of 1–20% with a median of 2.5% (1%, 9%) after surgery for functional corticotroph adenomas (*n* = 14 patients with available data). However, the proportion of patients with Ki-67 ≥ 3% increased from 22.2% (2/9) at the first surgery to 50% (7/14) at the time of diagnosis of a functional SCA.

In addition, Crooke's cell adenomas are more aggressive and invasive in clinical practice, with a high recurrence rate and the possibility of developing into pituitary cancer (11). Crooke's cell adenoma was occasionally found either in functional corticotroph adenomas or SCAs depending on the hyaline changes and the expression of circular low molecular weight keratin under pathological examinations. There was no Crooke's cell adenoma in this group of SCA patients after reviewing by pathology experts.

### Treatment

**Figure 2:**
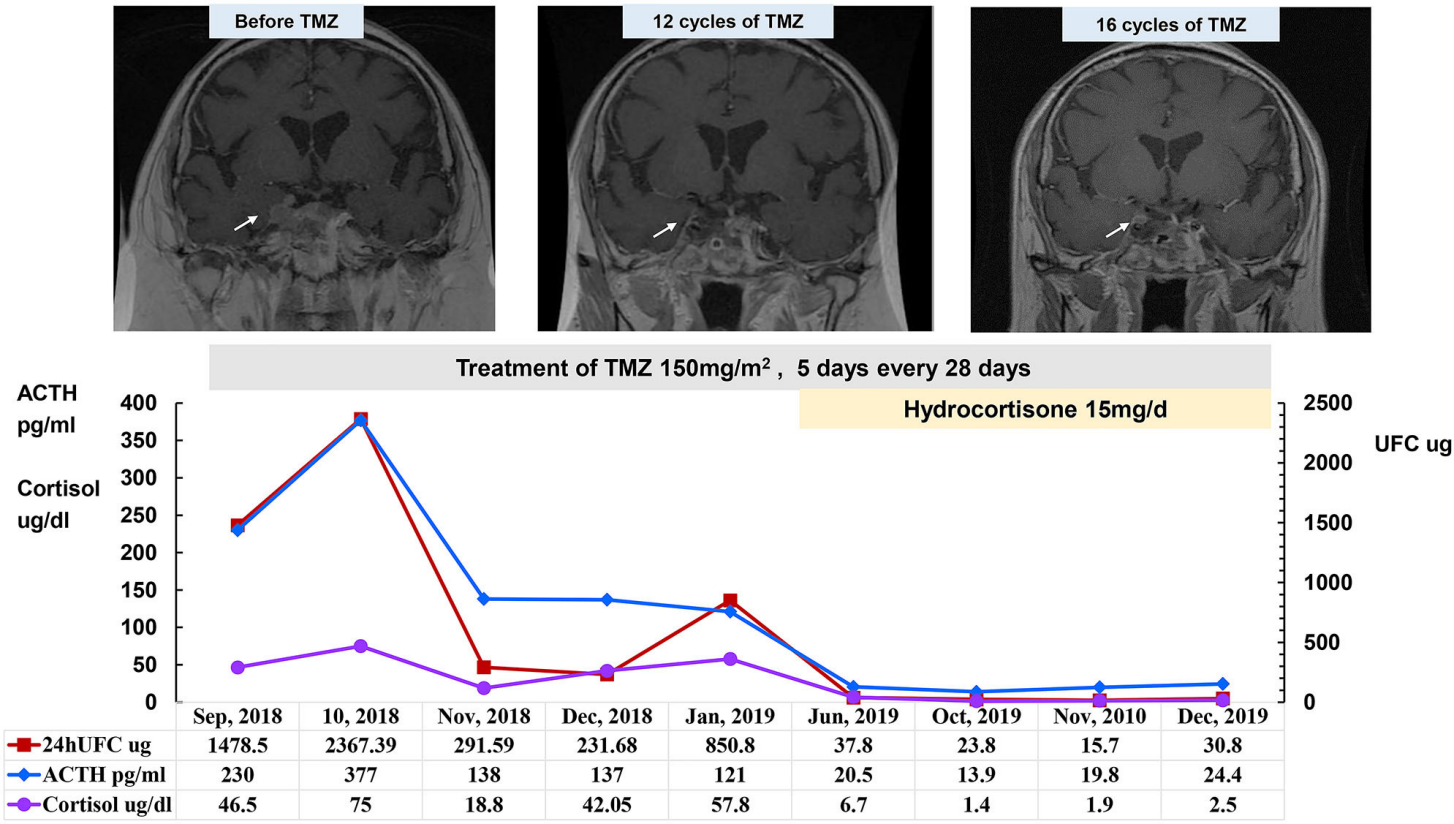
Changes in 24-h UFC, morning ACTH, and morning cortisol levels after treatment with TMZ after transformation to a functional SCA in case 2 (September 2018–December 2019). Patient in case 2 started TMZ in September 2018 after five surgeries and three courses of radiotherapy. Serum cortisol and plasma ACTH levels returned to within the normal ranges after treatment with TMZ for 12 cycles. Hydrocortisone was temporarily suspended on the day of the 24-h UFC collection in October, November, and December 2019. Prior to TMZ treatment, enhanced MRI showed irregular boundaries and an uneven texture of the tumor tissue that invaded the right cavernous sinus; the tumor tissue was significantly reduced after TMZ treatment.

Because the SCAs recurred frequently, surgery could only temporarily reduce the tumor size and relieve the compression symptoms. Even after several surgeries and adjuvant radiotherapy, the tumors often relapsed in a short time, which indicated that these cases were refractory pituitary adenomas. All 16 patients experienced one to five surgeries (median of 2.5 [2.0, 4.0] surgeries), which was much more than the surgeries needed for patients with common pituitary adenomas. Because most of the tumors were invasive macroadenomas, the tumor could not be completely removed by surgery. Thirteen patients received radiotherapy with remission in four cases (30.8%) and uncontrolled in nine cases (69.2%). In the patients who achieved remission, one patient underwent proton knife (case 3), and three underwent fractionated radiotherapy (cases 1, 6, and 7). In all four cases, the tumor volume shrank, and ACTH and cortisol were controlled within the normal range after radiotherapy with a follow-up time of 3–44 months. The other nine patients showed progression or recurrence after radiotherapy (five cases with fractionated radiotherapy and four cases with gamma knife). Because the treatment was so challenging, with quick recurrence and lost opportunity for surgery, five patients (cases 2, 5, 6, 13, and 15) were treated with temozolomide (TMZ). Case 6 developed extensive skin rashes after taking TMZ for 2 days; the rashes recovered after drug withdrawal and were aggravated after reuse; therefore, TMZ was discontinued. In addition, case 2 started TMZ (150 mg/m^2^ for 5 days every 28 days) in September 2018 after five surgeries and three courses of radiotherapy. The serum cortisol and plasma ACTH levels returned to the normal ranges, the tumor volume was significantly reduced after 12 cycles of TMZ, and adrenal insufficiency occurred after 13 cycles of TMZ. As of January 2020, this patient had completed 17 cycles of TMZ treatment, and the treatment was ongoing with a rather good therapeutic effect (Figure 2). Cases 13 and 15 were also treated with TMZ at 150–200 mg/m^2^ for 5 days every 28 days for 15 cycles and 11 cycles, respectively. These two cases also obtained both tumor volume reduction and normalization of ACTH and cortisol levels. However, ACTH and cortisol levels decreased significantly in case 5 after six cycles of TMZ treatment, whereas the tumor continued to progress. Finally, the patient in case 5 died of tumor progression that compressed the brain stem. Among the four patients with long-term therapy with TMZ, there were no significant adverse reactions, such as rash, gastrointestinal reaction, leukopenia, or abnormal liver function tests. Therefore, TMZ is a promising drug for the treatment of SCA that has transformed into clinically functional Cushing's syndrome.

### Follow-Up

The median follow-up time for these patients was 110.9 ± 85.3 months (24–357 months). Although surgery and radiotherapy could reduce the tumor tissue and relieve the compression symptoms caused by the tumor, recurrence and progression of the tumor occurred in a short time after the earlier mentioned treatment. After comprehensive treatment, 10 patients were in remission at the last follow-up. Eight patients remained in remission with both tumor and hormone control: one patient (case 11) underwent only one surgery with a follow-up time of 72 months; four patients (cases 1, 2, 3, and 7) underwent surgeries and radiotherapy with follow-up times of 357, 141, 112, and 115 months, respectively; and three patients (cases 6, 13, and 15) underwent surgeries, radiotherapy, and TMZ treatment with follow-up times of 81, 168, and 48 months, respectively. A reduction in the tumor without effective hormone control was observed in case 8 (after four surgeries and two courses of radiotherapy with a follow-up time of 57 months) and case 9 (after four surgeries with a follow-up time of 24 months). Two patients died. The patient of case 4 died of severe hydrocephalus due to rapid tumor progression, although he received three surgeries, one hydrocephalus shunt operation, one course of radiotherapy, and 3 months of 20-mg long-acting octreotide every month intramuscularly. The patient of case 5 died of progressive tumor compression of the brainstem, although he received radiotherapy, surgery, and six cycles of TMZ treatment.

## Discussion

SCAs account for 3 to 6% of all pituitary adenomas, 10 to 20% of silent pituitary adenomas, and ~40% of all corticotroph cell tumors (19–23). SCA was also the second most common tumor (16%) in NFPAs differentiated by combined IHC staining for ACTH and Tpit. Although the pathological results of SCAs showed positive expression of ACTH in most cases, the SCAs were usually diagnosed as NFPAs before the first surgery, as their clinical manifestations, laboratory examinations, and imaging were very similar to NFPAs. Most SCA cases remained “silent” for a long time. Very rare cases of SCAs have the potential to transform into clinically functional corticotroph adenomas (13–18). To investigate the clinical features of SCAs with transformation, a total of 16 SCAs with detailed data were collected from our medical center and the literature.

The sex ratio of males to females was 1:1.3 in this group of converted SCAs, which was much lower than that of common Cushing's disease (1:3–8) (24). The most common age group of patients with SCAs at the time of diagnosis of Cushing's syndrome was 30–49 years (11 cases, 68.8%), which coincided with the observed age range for other patients with Cushing's disease (mostly diagnosed at the age of 25–45 years). The typical interval from the initial visit to the diagnosis of a functional SCAs was 12–36 months (9/16, 56.3%), and the longest interval was 120 months. One study showed that the recurrence rate of SCAs was 31%, which is similar to that of NFAs (25), whereas, in our study, the recurrence rate of functional SCAs was 100%. As a result, SCA patients with repeated recurrence must be carefully monitored for the potential of conversion to a functional type. The long-term follow-up of evaluation of the HPA axis was required for recurrent SCAs.

It was suspected that even SCA microadenomas might have the chance to convert to clinical Cushing syndrome. However, in this group of converted SCAs, the tumors were all macroadenomas, and none were microadenomas. The reason might be that the diagnosis of SCA can only be initially confirmed by the pathological results with positive ACTH or Tpit immunostaining after surgery. SCA microadenomas were usually too small to be detected without the relevant symptoms of tumor-mass effects; thus, these patients may not have the indications for surgery or pathological examination. As a result, it was difficult to investigate the incidence of microadenomas among SCAs.

The pathogenesis of transformation of corticotroph adenomas from the silent type to the functional type is still unclear, but it was speculated that the biological activity of ACTH decreased in SCAs, which led to the fact that, although ACTH was IHC positive, the efficacy was not enough to cause biochemical and clinical symptoms of Cushing's syndrome (26). The other cause for the silent nature of ACTH among most SCAs has been demonstrated by lysosome dysfunction and the inability to destroy ACTH before it is secreted (20). Other theories include Golgi complex dysplasia leading to the mispackaging of ACTH (27, 28). In addition, PC1/3 is an enzyme that can cleave the precursor of ACTH, proopiomelanocortin (POMC), into ACTH. Whereas, overexpression of POMC has been found in SCA tumor tissues, the expression of PC1/3 was decreased; thus, POMC could not be broken down into enough ACTH to cause typical hypercortisolism (16, 29). Therefore, it might take a long time for ACTH to rise gradually to a higher level to cause clinical Cushing's syndrome. The time interval for transformation from the silent type to the functional type of SCA took a median of 30 months with the longest interval of 120 months, which possibly accounts for this speculation.

Patients with SCAs usually do not have symptoms of hypercortisolism before conversion; thus, their Cushing's syndrome remains silent (30). Patients often come for the first clinical visit because of intracranial compression symptoms, including visual disturbances, headache, and hypopituitarism, etc. Compared with a study reported by Adriana et al., the incidence rates of visual impairment were 39.4% (13/33) in SCAs and 46.8% (59/126) in NFAs (31), and visual impairment was more common in the functional SCAs in our study (10/16, 62.5%). After the appearance of functional corticotroph adenoma, the biochemical changes and hormonal changes seemed more serious. Frequent hypokalemia was observed in seven patients (100%), and it was often significantly higher than that in patients with common Cushing's disease (10–25.64%) (24, 32). In addition, the median increase values in morning ACTH, serum cortisol, and 24-h UFC in SCA patients were 3.8-, 1.7-, and 5.3-fold, respectively, above the ULN, respectively. In a study including 197 patients with common Cushing's disease, the average morning ACTH (72.90 pg/ml), morning cortisol (28.13 μg/dl), and 24-h UFC (474.90 μg) were increased 1.6-, 1.5-, and 3.7-fold, respectively (32). As a result, the decrease in potassium and increase in ACTH and 24-h UFC in functional SCAs were more significant than those in common Cushing's disease. As we know, microadenomas accounted for most cases of Cushing's disease, whereas all SCAs that transformed to functional types were macroadenomas initially, and the tumor sizes became larger after conversion. In general, it was thought that the larger tumors caused the stronger ability of hormone production. However, before conversion to Cushing's syndrome, the SCAs had already been macroadenomas without overproduction of hormones; therefore, the mechanism that triggered the tumor conversion to produce more hormones still needs to be investigated. In addition, five functional SCA patients underwent high-dose DST with a paradoxical increase in three cases, which is quite different from the suppression in ~80% of cases that were typically observed in Cushing's disease (24). The high-dose DST was not mentioned in the other nine cases from the relevant literature, so it was impossible to know whether they had the same characteristics. This result suggested that the function of SCA tumors was more autonomic and that it was not easily suppressed by dexamethasone. Raverot et al. observed that following intravenous dexamethasone, the 24-h UFC and nadir ACTH levels were significantly higher in the corticotroph macroadenoma group than those in the microadenoma group. A subsequent molecular study revealed that there were no differences among the SCA, macroadenoma, and microadenoma groups in terms of the level of glucocorticoid receptor α or messenger RNA expression. Therefore, resistance to glucocorticoid suppression could not be explained by changes in glucocorticoid receptor α expression in SCAs (33).

In the 16 patients in this study, the tumors not only were macroadenomas but also had obvious invasiveness. Altogether, 68.8% of cases in this study had cavernous invasion, which led to the difficulty of complete removal of the tumor and possible residual tumor progression or recurrence after surgery. In addition, in five cases with available tumor sizes both initially and at the time of functional SCA diagnosis, the tumor volume increased from 1.7–29.2 to 3.2–30.8 cm^3^. The tumor volume increased 1.71 ± 0.6-fold (1.0- to 2.65-fold) from the initial non-functional SCA to the transformation to a functional SCA for each patient. This suggested that the tumor had more progression than previously when it converted to a functional SCA. Although the value of Ki-67 in predicting tumor aggressiveness remains controversial (34–36), the proportion of patients with Ki-67 ≥ 3% reached 50% and was consistent with the larger tumor volume at the time of functional SCA diagnosis (11, 34). The other fact worth noting is that the cystic changes were found on pituitary MRI in 50% (7/14) of the functional SCA patients. The MRI images suggested that the cyst changes were macrocystic, whereas the lack of a description of T2-phase images in most patients made it impossible to assess further microcystic changes. Langlois et al. reported a case series of SCAs (*n* = 39), and 33% of cases demonstrated >50% fluid content on MRI T2-phase sequences (37). Cazabat et al. reported that cystic changes could be found in all SCAs (*n* = 17), half of corticotroph macroadenomas (*n* = 14), and only 17% of silent gonadotroph macroadenomas (*n* = 60) on T2-weighted pituitary MRI sequences in their case series (38). Multiple microcysts were present in most SCAs (13/17, 76.5%), and macrocysts were observed in the remaining four SCAs (4/17, 23.6%). In contrast, the specific finding of multiple microcysts was present in only 5% of silent gonadotroph macroadenomas. The presence of multiple microcysts had a sensitivity of 76% and a specificity of 95% for predicting an SCA (38, 39). Kasuki also found that microcystic changes were more common in SCAs (7/12, 58.3%) than in other pituitary tumors (6.9%) and had a sensitivity of 58%, a specificity of 93%, and an accuracy of 90% for defining an SCA (40). In this study, cystic changes, mostly macrocystic changes, were found in 50% of the functional SCAs (7/14) on pituitary MRI. Therefore, we should pay attention to SCA patients with macrocystic changes on MRI who may have the possibility of a transformation of functional type.

SCA, which can sometimes transform into functional Cushing's syndrome, is not only a rare disease but also a kind of refractory pituitary adenoma. During the disease, patients often underwent several surgeries and radiotherapy, but the tumor recurred ~1–3 years after treatment, and some patients did not have the possibility of reoperation. Therefore, the choice of radiotherapy and medical treatment became more important for adjuvant therapy. In total, 13 patients received radiotherapy, and only four patients (30.8%) achieved a remission. However, the remission rate of radiotherapy in common Cushing's disease was 66.7–83% (41, 42).

The European Society of Endocrinology survey from 2016 reported that for patients with failed surgeries or radiotherapy, TMZ was still effective in some aggressive pituitary adenomas with a remission rate of 37% (*n* = 156) (43). In addition, clinically functioning pituitary tumors were more likely to demonstrate regression on TMZ treatment compared with non-functioning tumors (45 vs. 17%, *P* = 0.01). Furthermore, there was no clear difference in efficacy among pituitary tumors with different subtypes of function. According to the subtype of pathology, the most effective response to TMZ was lactotroph adenomas (50%), followed by somatotroph adenomas (43%), corticotroph adenomas (38%), thyrotroph adenomas (25%), and gonadotroph adenomas (17%) (44). Four patients in our study were treated with TMZ for a long time, and three patients achieved long-term hormone control and tumor control with a treatment duration of 6–17 cycles. The additional one patient with TMZ treatment had been under hormone control, but the tumor continued to progress and caused death. The relatively preferable effective result of TMZ treatment was achieved in three patients in our group, probably because the tumors had become functional when receiving TMZ treatment. To date, TMZ has been a very helpful and well-tolerated treatment for patients with functional SCA.

In general, SCAs that have completely transformed into functional SCAs are quite rare. Limited cases have been collected to investigate their clinical features. SCAs that can transform into functional SCAs tend to be macroadenomas with a more frequent invasion of the cavernous sinus and higher recurrence, even after multiple steps of treatment. The tumor volume became larger at the time of diagnosis of a functional SCA, combined with an increase in the Ki-67 index. After the diagnosis of Cushing's syndrome, the ACTH and 24-h UFC increased, and the potassium decreased more significantly than in common Cushing's disease. In addition, macrocystic changes in pituitary MRI were observed in half of this group of SCA patients. During treatment, surgery was able to remove the resectable tumor tissues and decompress the oppression symptoms. Radiotherapy was helpful to nearly 1/3 of patients but led to a lower remission rate than in patients with common Cushing's disease. TMZ is a well-tolerated and promising adjuvant medical treatment with good efficacy in patients with functional SCAs. Above all, although it is rare, long-term follow-up is required to monitor the occurrence of hypercortisolemia once a clinically non-functional SCA is diagnosed.

## Data Availability Statement

All datasets presented in this study are included in the article.

## Ethics Statement

The studies involving human participants were reviewed and approved by the Ethics Committee of Peking Union Medical College Hospital Peking Union Medical College Hospital, Chinese Academy of Medical Science and Peking Union Medical College. Written informed consent for participation was not required for this study in accordance with the national legislation and the institutional requirements.

## Author Contributions

GZ wrote the main manuscript text and prepared the figures and tables. LL, HZhu, HY, MF, XL, CD, YY, RW, HZha, XS, and ZL collected and analyzed the data. LL and ZL designed the study and critically revised it for important intellectual content. All authors reviewed the manuscript and approved it for publication.

## Conflict of Interest

The authors declare that the research was conducted in the absence of any commercial or financial relationships that could be construed as a potential conflict of interest.
